# Rare Presentation of Primary Extramedullary Plasmacytoma as Lip Lesion

**DOI:** 10.1155/2017/4296802

**Published:** 2017-03-16

**Authors:** Mali Him, Maggie Meier, Vikas Mehta

**Affiliations:** ^1^Mount Sinai Hospital, Chicago, IL, USA; ^2^St. Luke's Hospital St. Louis, MO, USA

## Abstract

Malignant plasma cell proliferation can be presented as part of disseminated disease of multiple myeloma, as solitary plasmacytoma of bone, or in soft tissue as extramedullary plasmacytoma. Extramedullary plasmacytomas represented approximately 3% of all plasma cell proliferation. Approximately 80% of extramedullary plasmacytomas occur in the head and neck region while the other 4% occur in the skin and to a lesser extent in the lip. In this paper, we report a rare case of primary cutaneous plasmacytoma involving the lip in a 65-year-old male. The patient presented with a nonhealing lower lip sore for the past 3 years. Upon further workup, there was no evidence of multiple myeloma or light chain disease. The patient was treated with radiation therapy and his last follow-up revealed no evidence of multiple myeloma or light chain disease.

## 1. Introduction

A plasmacytoma is a tumor of plasma cells which often develops as a systemic spread of multiple myeloma, in bone as a solitary plasmacytoma of bone, or in soft tissue as extramedullary plasmacytoma (EMP). Extramedullary plasmacytomas are rare, comprising about 3% of all plasma cell neoplasms with bone marrow plasma cell infiltration <5% of nucleated cells and demonstrating no evidence of myeloma [1]. While solitary bone plasmacytomas reveal single tumor inside the bone comprising of abnormal plasma cells. The majority (60%–80%) of extramedullary plasmacytomas are found in the head and neck region, particularly in the upper respiratory tract, and to a lesser extent in gastrointestinal tract. Cases of primary cutaneous plasmacytoma are comprised of 2–4% of extramedullary plasmacytomas. It is unusual to have a primary cutaneous plasmacytoma present as a lip lesion, as only 5 cases have been reported in the literature [2]. The first case reported was by Volk in 1936 [2].

Primary cutaneous plasmacytomas present as relatively slow growing papules or plaques or an erythematous-violaceous nodule either existing in solitary tumor (62%) or possibly involving multiple sites (38%). Patients usually present with a benign past medical history. Symptoms are dependent on location and size of lesion. Rarely, patients present with fever, malaise, night sweats, or weight loss. Other features that may be present are lymphadenopathy and hepatosplenomegaly. The lesion may be ulcerated and necrotic. The skin lesions range in diameter from 1 to 5 cm and the shape may vary. Histologically, it shows a nonepidermotropic dermal infiltrate of plasma cells at different stages of maturation. The plasma cells often show atypical, binucleation, and increased mitotic activity. The epidermis is generally spared. The mean age at diagnosis is 60 years with a male to female predominance of 3 : 1 and it is commonly reported in Asian men. The incidence rate increased with advancing age.

Treatment for solitary or a few lesions can be treated with radiotherapy or surgical excision. Chemotherapy or intralesional corticosteroid therapy may be provided for multiple skin lesions.

Due to this rarity, we feel the need to present this case in order to increase the awareness of the disease to better serve our patients. The clinicopathological features, prognostic factors, and treatment options with its unusual presentation of lip involvement is being discussed in this report.

## 2. Case Description

A 65-year-old male presented to the Ears, Nose, and Throat clinic in September 2015 with complaints of a nonhealing lower lip sore for the past 3 years. The slow growing lesion was described as a burning sensation with scabbing and occasional bleeding. It was slowly increasing in size. The patient had a greater than 50-year history of smoking tobacco (not chewing) which he quit about 7 months prior to his visit. He also complained of unintentionally losing 3 lbs in the last 2 weeks. He denied any fevers or chills. He had no other complaints.

Physical examination was significant for a single lesion on the lower lip 1.5 × 1 cm. A biopsy was performed and sent to pathology. The patient was worked up thoroughly for evidence of multiple myeloma/light chain disease. Laboratory testing was significant for no anemia, normal serum calcium, normal serum albumin, normal total protein, and normal creatinine. There was no Bence Jones protein in urine. Bone survey was done which was negative. Bone marrow biopsy showed <5% plasma cells, ruling out plasmacytoma of the bone. No monoclonal gammopathy was seen, which precluded immunofixation order. Kappa to lambda ratio was normal.

**Figure 1:**
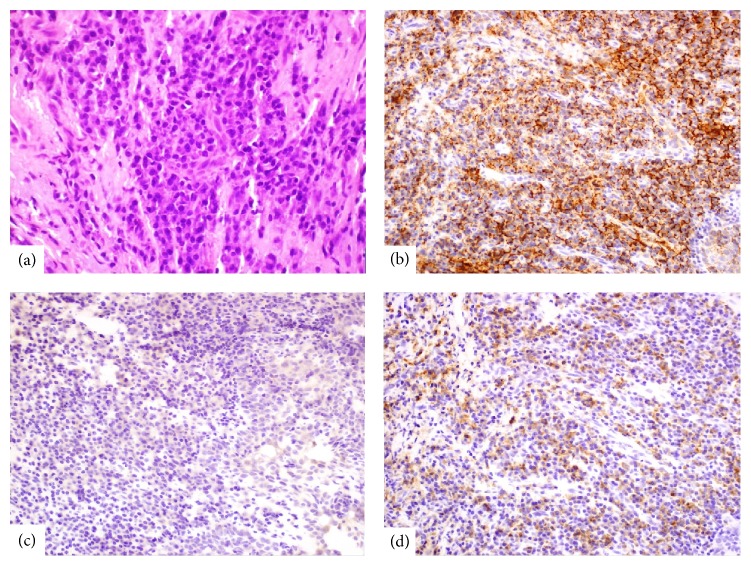
(a) Dense plasma cell infiltrate, H&E, 40x; (b) CD138 immunostain labeling plasma cells, IHC 40x; (c) Kappa in situ hybridization with only rare plasma cell labeling, ISH, 40x; (d) Lambda in situ hybridization with most plasma cells labeling, ISH, 40x.

Hematoxylin-eosin staining of the lip biopsy revealed a dense plasmacytic infiltration in the deep dermis (see Figure 1(a)). Immunohistochemical panel included CD3, CD20, and CD138 immunohistochemical stains as well as kappa and lambda light chain in situ hybridization. The superficial dermis and the epidermis were not seen to be involved. Immunohistochemistry was positive for CD138 (see Figure 1(b)) and revealed that most plasma cells were lambda positive and with rare kappa-positive plasma cells (see Figures 1(c) and 1(d)).

Based on the ISCL/EORTC Proposed on TNM Classification of Cutaneous Lymphoma our patient was staged at T1aN0M0. The patient was treated in November 2015 with radiation therapy as follows: 3000 cGy in 15 fractions using 3D conformal technique to his lower lip and level I lymph nodes and he received 2000 cGy boost bringing his total dose to 5000 cGy in 25 fractions. He responded well to treatment. At his last follow-up, there was no evidence of multiple myeloma or light chain disease.

## 3. Discussion

Primary cutaneous plasmacytoma is a rare diagnosis that represents approximately 4% of extramedullary plasmacytoma cases. Of the 4% cases, only a few cases have been reported to be associated with lip lesions. In the latest comprehensive literature review by Tsang et al. in 2016, there are 5 case reports of primary cutaneous plasmacytomas of the lip [2]. Primary cutaneous plasmacytomas without underlying multiple myeloma is included in the 2005 WHO/EORTC classification among the primary cutaneous marginal zone B-cell lymphoma due to considerable overlapping features [3]. Primary cutaneous marginal zone B-cell lymphoma is considered as part of extranodal marginal zone lymphoma of MALT type [3]. Due to the infrequent and nonspecific presentation of cutaneous plasmacytomas, a clinical diagnosis is not sufficient. Diagnosis is based on clinical, histopathological, and immunohistochemical findings, and multiple myeloma must be ruled out by laboratory, radiologic, and bone marrow studies [4]. After ruling out multiple myeloma, the only findings will be a histopathologic dense monomorphic dermal plasmacytic infiltrate and a monoclonal immunohistochemistry.

Consistent with published literatures, our male patient in this case, at the age of 65, presented with a slow growing lesion and insignificant systemic review. Tissue biopsy immunohistochemically shows cytoplasmic expression of immunoglobulins that are restricted to one immunoglobulin chain. Histopathologic finding of diffuse or nodular dermal and subcuticular infiltrate of plasma cells, displaying varying degrees of maturation and atypia but no epidermotropism, are findings that are consistent with our patient. The immunophenotype demonstrates plasma cells positive for CD138, CD38, and CD79a, but generally not CD20 and leukocyte common antigen, and shows monotypic cytoplasmic immunoglobulin light chain expression on paraffin sections [3]. On the other hand, cutaneous marginal zone B cells express CD 20. Our patient was positive for CD138 and negative for CD20. While 30% of all marrow-based myelomas are lysozyme positive, lysozyme is not expressed by cutaneous plasmacytomas.

Laboratory, radiological, and bone marrow investigations must be done to exclude multiple myeloma. Laboratory studies include but are not limited to complete blood count, blood smear, blood sedimentation rate, renal function, electrolyte (calcium), serum and urine protein electrophoresis, quantitative Ig determination in serum, immunoelectrophoresis in serum and urine, and B-2 microglobulin determination in serum. All laboratory findings will be normal except for quantitative Ig elevation. Radiological studies include a full skeletal X-ray to rule out any osteolytic lesions that are consistent with multiple myeloma. A bone marrow biopsy will demonstrate plasma cell infiltration less than 5% of all nucleated cells. Plasma cells infiltration is common in reactive lesion and must be ruled out with staining for kappa or lambda light chain. In contrast to multiple myeloma, extramedullary plasmacytoma showed absence of cyclin D1 and infrequent expression of CD56.

Other differential diagnoses included secondary cutaneous plasmacytoma in the setting of systemic disease, mucosal extramedullary plasmacytoma with secondary skin involvement, marginal zone B-cell lymphoma, plasma cell granuloma, non-Hodgkin lymphoma, high-grade malignant immunoblastic lymphoma, and poorly differentiated neoplasms, as well as infectious diseases that cause infiltration of plasma cells.

Currently, there are approximately 50 cases of primary cutaneous plasmacytomas that have been reported in the literature [2]. Due to its rarity, little is known about its treatment. Treatment options include surgery, radiation, chemotherapy, and intralesional corticosteroid therapy. Type of treatment depends on the numbers (solitary versus multiple) and location of lesions. Most patients with solitary primary cutaneous plasmacytoma received radiotherapy as a component of treatment. Radiotherapy with curative intent (>4000 cGy) is the standard of care and provides promising result since the tumor is radiosensitive. The optimal dose for local control is 40–50 Gy in 20 fractions delivered over 4–6 weeks. However, caution should be considered when using radiotherapy as local recurrence or lymph node metastases could develop thereafter [2]. In addition to high dose radiotherapy and fractions, adjuvant chemotherapy may also be considered for lesions with high-grade histology or tumors >5 cm or for patients with multiple lesions. Palliative irradiation may also be used for the most symptomatic and large lesions. At the time when this case was written, the patient was in remission with no evidence of multiple myeloma or light chain disease. As reported in previous literatures, patient with solitary lesions received better outcomes than patient with multiple lesions at presentation. They tend to have less local regional or distant dissemination and remained confined to the skin. As a note, large solitary lesions might have a higher risk of progression to metastatic disease, although a size cut-off had not been determined [2].

The main prognostic factor seems to be the clinical presentation with more aggressive cases associated with multiple lesions, larger size, and IgA secretion by neoplastic cells. Multiple myeloma has been estimated to develop in one-third of patients [5]. It is found that progression to multiple myeloma is lower in soft-tissue plasmacytomas compared to osseous plasmacytomas [2]. Patients may also develop local recurrence and distant skin recurrence. Patients with multiple lesions had a higher likelihood of progression to multiple myeloma and subsequent death [2]. From previous case reported, recurrent-free survival increases from 1.4 years to 11 years and overall survival rate increases from 4.3 years to 17 years in patients with solitary primary cutaneous plasmacytomas compared to those with multiple primary cutaneous plasmacytomas lesions [2].

Currently, the pathophysiology is unknown; however there are a few developing hypothesis. Of all up to date cases of primary cutaneous plasmacytoma, five have been described in which local triggering stimulus from trauma may have contributed to the development of plasma cell proliferation. One of these cases reported a primary cutaneous plasmacytoma localized to the lower lip, which had been affected with recurrent herpes simplex virus-1 for 15 years. It was postulated that chronic stimulation of keratinocytes by herpes simplex virus-1, possibly through toll-like receptors, may have favored the release of interleukin-6 able to induce plasma cell proliferation, transformation, and survival [6]. Interleukin-6 may also have some oncogenic effects via enhancement of C-MYC and is antiapoptotic [7]. In the second hypothesis, it is thought that plasma cells expressing CXCR4 cytokine receptor can interact with the inflammatory cascade released due to tissue injury, facilitating the migration of neoplastic plasma cells into the skin [8].

Genetically, extramedullary plasmacytomas share many features of other plasma cell disorders, such as recurrent losses in chromosome 13, chromosome arm 1p, and chromosome arm 14q, as well as gain in chromosome arms 19p, 9q, and 1q [9].

According to the International Society for Cutaneous lymphoma (ISCL) and the cutaneous lymphoma task force of the European Organization of Research and Treatment of Cancer (EORTC), TNM classification system (see the list below) had been proposed to primarily document disease extension and to a lesser extent for prognostic guide [10].

*ISCL/EORTC Proposed TNM Classification of Cutaneous Lymphoma Other Than Mycosis Fungoides and Sezary Syndrome*

(T) *⁡*
  (T1) Solitary skin involvement
    (T1a) A solitary lesion <5 cm diameter
    (T1b) A solitary >5 cm diameter
  (T2) Regional skin involvement of multiple lesions limited to 1 body region or 2 contiguous body regions
    (T2a) All-disease encompassing in a <15 cm diameter circular area
    (T2b) All-disease encompassing in >15 and <30 cm diameter circular area
    (T2c) All-disease encompassing in a 30 cm diameter circular area
  (T3) Generalized skin involvement
    (T3a) Multiple lesions involving 2 noncontiguous body regions
    (T3b) Multiple lesions involving equal to or greater than 3 body regions
(N) *⁡*
  (N0) No clinical or pathologic lymph node involvement
  (N1) Involvement of 1 peripheral lymph node region that drains an area of current or prior skin involvement
  (N2) Involvement of 2 or more peripheral lymph node regions or involvement of any lymph node region that does not drain an area of current or prior skin involvement
  (N3) Involvement of central lymph nodes
(M) *⁡*
  (M0) No evidence of extracutaneous non-lymph node disease
  (M1) Extracutaneous non-lymph node disease present

In order to stage the disease, patients should undergo the following testing: a complete history and physical examination, complete blood count with differential count, chemistry panel with LDH, flow cytometry on peripheral blood, and imaging with computed tomography scan of the chest, abdomen, and pelvis with contrast or positron emission-tomography/CT as it can visualize metabolically active disease in relatively small lesions [10].

According to this classification, our patient was stage T1aN0M0 disease. As for treatment management, clinicians are recommended to follow the WHO-EORTC cutaneous lymphoma classification to understand the clinical behavior and prognostic variables in the heterogeneous group of cutaneous lymphomas in order to provide optimal care. Due to its shortcomings, the proposed TNM system will be reexamined as more data are obtained.

## 4. Conclusion

With few cases in the literature reported, this case is an example of a primary cutaneous lesion in an unusual location with no systemic disease. Follow-up care includes continual monitoring for progression to multiple myeloma. This consists of M-protein measurements and complete blood counts at six-week intervals for the first 6 months. This rarer and nonspecific presentation of a primary extramedullary plasmacytoma is important to keep in mind when evaluating lip lesions.
